# Successful Tenckhoff catheter salvage in a patient with peritoneovesical fistula: A case report 

**DOI:** 10.5414/CNCS109656

**Published:** 2019-04-16

**Authors:** Vamsikrishna Makkena, Varun Kumar Bandi, Deepashree G. Anandkumar, Renuka Prasad Yelahanka, Manikantan Shekar, Ramprasad Elumalai, Jayakumar Matcha

**Affiliations:** 1 Department of Nephrology, Sri Ramachandra Medical College, Chennai,; 2 Department of Nephrology, Dr. Pinnamaneni Siddhartha Institute of Medical Sciences & RF, Gannavaram, Andhra Pradesh,; 3 Apollo Hospitals, and; 4 Fortis Hospitals, Bangalore, India

**Keywords:** PD catheter removal, peritoneal dialysis, peritoneol vesical fistula

## Abstract

Introduction: Many techniques are available for inserting peritoneal dialysis (PD) or continuous ambulatory peritoneal dialysis (CAPD) catheters, with varying possible complications. We report a case of bladder perforation that was managed with catheter salvage. Case report: A 48-year-old man with end-stage renal disease (ESRD) underwent CAPD catheter placement percutaneously, with tip in the pelvis. On the 3^rd^ day after placement, the patient complained of increase in urinary volume with PD flushing. Urine analysis showed 3(+) glucose and absent creatinine. Cystogram showed the catheter abutting the bladder wall. CT of the abdomen showed the catheter piercing the bladder and exiting through the posterior wall. The PD catheter was repositioned under fluoroscopy. Discussion: The complications surrounding insertion of CAPD catheter can be either mechanical or infectious. Peritoneo-vesical fistula or placement of the PD catheter into the urinary bladder is a very rare complication. The possibility of catheter salvage should be entertained while discussing management options.

## Introduction 

Peritoneal dialysis (PD) or continuous ambulatory peritoneal dialysis (CAPD) is one of the modalities of dialysis advised for end-stage renal disease (ESRD) patients. There are many techniques for inserting CAPD catheters, including open surgical, laparoscopic, blind percutaneous or peritoneoscopic or fluoroscopy-guided insertion. Back when this modality was first initiated, the open surgical method was the most preferred option. In recent years, an increasing number of practitioners are preferring the percutaneous or the laparoscopic approach [1, 2, 3]. At our center, the percutaneous procedure (using 16G needle, guidewire, and 16F peel away sheath/dilator) is the usual preferred option, with the advantage of lower economic burden to the patient and a shorter postprocedure hospitalization with reduced morbidity. However, the disadvantage of the technique lies in the fact that the peritoneal cavity is not directly visualized. This drawback had been shown to be associated with slightly higher mechanical complications. Malfunctioning due to migration of the catheter tip, kinking, and obstruction due to fibrin deposition or a clot in the catheter are the main mechanical complications reported in the literature [1]. Perforations of the nearby organs are rare, and very few have been reported [1, 4]. We report a case of bladder perforation in a patient who underwent a bedside blind Tenckhoff catheter placement. The catheter was repositioned using fluoroscopy with catheter salvage and the bladder perforation was treated conservatively. There have been reports of bladder perforation with PD catheters, but this approach to the repositioning is the first such case described as per our knowledge. 

## Case report 

A 48-year-old man with ESRD secondary to diabetic nephropathy was admitted for initiation of renal replacement therapy. He had undergone 36 cycles (36 hours of 1 L/h cycle) of acute PD, through a temporary PD catheter inserted through the infra-umbilical approach, at another center 10 days prior and presented to us for maintenance dialysis. After discussing the options, he was started on hemodialysis through a temporary catheter and was planned to start on CAPD. He had a history of diabetic retinopathy and neuropathy. He did not have any history or symptoms suggestive of neurogenic bladder. 

**Figure 1.**
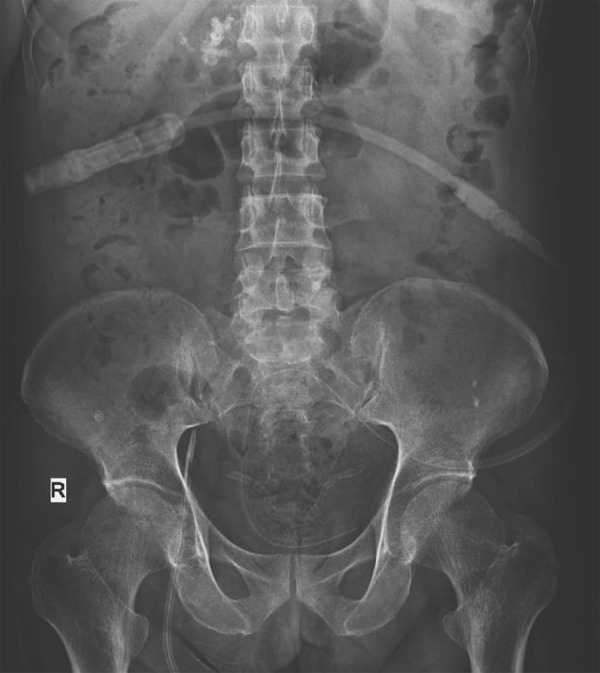
Post-CAPD catheter insertion abdominal X-ray confirming the tip position in the pelvis.

The patient underwent 3 sessions of hemodialysis and was planned for CAPD catheter insertion with vancomycin prophylaxis. As per protocol, he was advised to void his bladder completely and to pass stools before insertion. An infraumbilical midline incision was made under local anesthesia and IV sedation, an 18-G introducer needle was inserted through the rectus sheath until it reached the peritoneal cavity, and 1.5 L of peritoneal dialysate was instilled intraperitoneally. After instillation of fluid, a guidewire was passed through the needle, with the tip directed towards the suprapubic region. The needle was removed, the tract was dilated using a 16 Fr dilator followed by insertion of a 16 Fr peel away sheath/dilator. The guidewire was removed followed by the dilator, and Tenckhoff swan-neck double-cuff straight catheter was inserted through the peel away sheath. The sheath was peeled away, and a subcutaneous tunnel was created with the exit site facing down and out towards the left iliac fossa. The deep cuff was positioned over the peritoneum, and the superficial cuff was placed in the tunnel. The incision site was sutured in layers. Postprocedure, the inflow and outflow were found to flow without resistance, and the catheter was brought out through a left-sided tunnel. A 500-mL flush was given, and good inflow and outflow were noted. The outflow was initially blood-mixed, followed by drainage of clear fluid. There were no complications during the immediate postoperative period. With a plain abdominal radiograph, the position of the catheter tip was confirmed to be in pelvis (Figure 1). Ultrasonography was not used during the procedure due to logistic reasons. 

On the 3^rd^ day after insertion, after instillation of 500 mL of PD fluid, the patient complained of urinary urgency with an increase in urinary volume to 700 – 800 mL/day from his baseline of 300 – 400 mL/day. Also, there was decrease in the outflow volume with drainage of only 100 mL following instillation of 500 mL of PD fluid. With the increase in the inflow volume, there was a significant increase in his urine volume. Urine analysis was done to look for glycosuria, which showed a 3(+) on dipstick, from his baseline glycosuria of 1(+). He was asked to void after instilling 500 mL PD fluid through the PD catheter, and urinary creatinine testing was done, which was found to be absent. A possibility of peritoneovesical fistula was considered, and a cystogram was planned. The instilled fluid presumably entered predominantly into the urinary bladder through the side holes and led us to presume that the PD catheter could be in the preperitoneal location. Urine culture and PD fluid cultures were sent, which revealed growth of enterococcus (> 10^5^ colony forming units/mL), and he was treated with vancomycin intraperitoneally (IP) and broad-spectrum antibiotics intravenously. 

**Figure 2.**
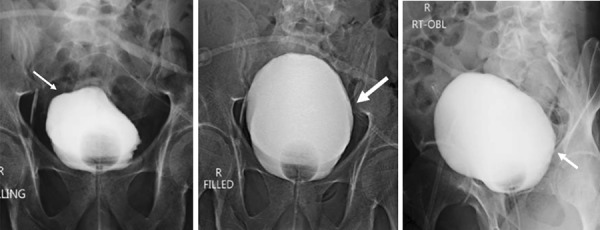
Cystogram showing the PD catheter abutting the urinary bladder. No evidence of bladder leak.

**Figure 3.**
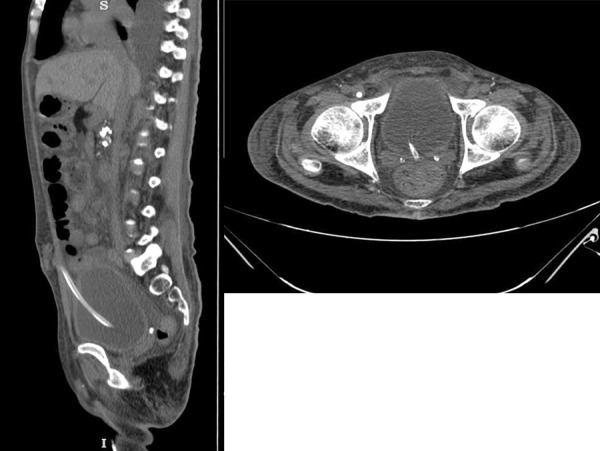
Plain CT abdomen demonstrating the PD catheter perforating the bladder anteriorly and exiting through the posterior surface.

The cystogram revealed a smooth-walled bladder, with no intraperitoneal leak of the contrast, and the catheter abutting the bladder wall (Figure 2). A plain abdominal computed tomography (CT) was performed to confirm the position (Figure 3). The catheter was seen to be piercing the anterior abdominal wall and entering the bladder anteriorly, perforating and coursing through the bladder before exiting through the posterior wall, with the tip in the rectovesical fossa. PD was discontinued, and he was continued on hemodialysis through a temporary femoral access catheter. After discussion with the urologists, it was planned to remove the catheter, followed by reinsertion at a later date, with conservative management for the bladder perforation. With cessation of PD, there was decrease in urine output to ~ 50 – 100 mL/day, and a repeat urine routine performed 3 days later showed reduction in pyuria (from plenty of white blood cells/high power field to 6 – 8 WBC/HPF). 

The options were discussed with the patient, and the need for a vascular access was explained. However, due to logistic reasons, an attempt was planned towards catheter salvage. He was planned for catheter repositioning using fluoroscopic guidance under the cover of intravenous and intraperitoneal broad-spectrum antibiotics. 

**Figure 4.**
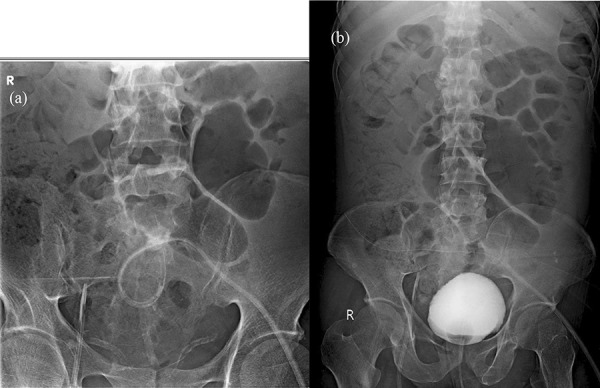
Fluoroscopic image showing the PD catheter being pulled back out of the bladder, with contrast through catheter tracking along the intestinal walls and no bladder filling (a) followed by repositioning of the tip in the right iliac fossa, away from the bladder with no evidence of bladder leak (b).

Using fluoroscopy with contrast (urograffin), first the bladder perforation was confirmed. The infraumbilical incision was re-explored under local anesthesia, while leaving the tunnel in situ. The deep cuff was identified, and the PD catheter was pulled back by ~ 6 cm (as measured by the prior abdominal CT image). Contrast was injected through the catheter, and it was confirmed to be intraperitoneal with no filling up of the urinary bladder. Using a guide wire, the catheter was redirected towards the right iliac fossa, and the position confirmed. On subsequent contrast injection through the PD catheter, the intraperitoneal nature and absence of bladder filling was confirmed. The bladder was later filled up with contrast, and there was no presence of any leaks (Figure 4). The initial bladder perforation could have been an intraperitoneal perforation since pulling back and re-positioning the catheter, without any new entry into the peritoneum, confirmed it to be in the peritoneal cavity. 

The patient was continued on IV antibiotics for 5 days, IP ceftazidime for a week, and IP vancomycin for 21 days, and was continued on hemodialysis. IP vancomycin was given in a single dwell (1,000 mL) for 6 hours every day. One week post repositioning, he was started on low-volume CAPD and discharged, and a repeat urine and peritoneal fluid culture were sterile. He was continued on Foley’s catheter for 4 weeks, with a residual urine output of ~ 200 – 300 mL/day. At 9-month follow-up, the patient is comfortable with CAPD 3 exchanges/day with good ultrafiltration (~ 1.4 L/day), and no change in urinary volume, with no further episodes of peritonitis. 

## Discussion 

The complications of insertion of CAPD catheter can be either mechanical or infectious [3]. The common mechanical complications are catheter obstruction, malfunction or malposition, peritoneal fluid leakage, hernias, and rarely abdominal organ injury [2, 3]. Peritoneovesical fistula or placement of the PD catheter into the urinary bladder is a very rare complication of CAPD catheter insertion, and very few cases have been reported [2, 5, 6, 7, 8, 9]. This complication can be easily avoided if the bladder is emptied before the procedure. Also, the diagnosis may be sometimes delayed due to misinterpretation of the radiographs taken. The diagnosis should be strongly suspected from the history and should be confirmed by chemical study of the extravasating fluid and by the demonstration of the fistula using contrast study. 

A literature review shows that bladder perforation occurs more commonly when using straight catheters, with no cases being reported with coiled catheters. There was no significant difference in the incidence of bladder perforation among the different percutaneous approaches. The complication occurs more frequently in diabetics [2], and could be possibly related to the presence of underlying diabetic neurogenic bladder or cystopathy. Another important risk factor includes history of prior abdominal surgery with presence of peritoneal adhesions. Our patient had a history of long-standing diabetes, and the possibility of incomplete voiding could have been a risk factor. Also, our patient had undergone acute PD 1 week prior, and whether this could have been a risk factor by promoting adhesions and increasing the risk of organ perforations has not been validated. 

The treatment of perforations of the urinary bladder is controversial, and also depends on the type of injury. Extraperitoneal ruptures (EPR) are generally managed conservatively, while intraperitoneal ruptures (IPR) are usually treated surgically. However, there is increasing evidence on conservative management of IPR in the absence of associated injuries or large perforations and sepsis and maintaining continuous bladder drainage [10, 11]. In our case, it could have been an IPR in view of the final position in the peritoneal cavity after the catheter had been pulled back. However, despite being an IPR, the bladder perforation was successfully managed conservatively. 

The reported cases suggest removal of the catheter with fistula closure followed by a reinsertion of PD catheter at the same sitting or a repeated sitting. In a case reported by Cornelis et al. [12], conservative management of the peritoneovesical fistula was attempted, but due to worsening peritonitis, the catheter was removed. To our knowledge, there are no cases reported of catheter salvage following a peritoneovesical fistula. Also, this could be the first reported case of successful catheter salvage and repositioning using fluoroscopic guidance under local anesthesia following a peritoneovesical fistula. 

The present case emphasizes the importance of complete evaluation of the patient preoperatively. It would be advisable to insert a Foley’s catheter and completely empty the urinary bladder to reduce the risk of bladder perforation, or a hand-held ultrasound could be used to confirm the urinary bladder volume. Also, the possibility of catheter salvage should be entertained while discussing management options, and performing the same under local anesthesia is a cost effective option, with reduced postprocedure morbidity and hospitalization. 

## Acknowledgment 

We acknowledge the Department of Radiology, Sri Ramachandra Medical College, for their contribution in the investigations. 

## Funding 

The authors have not received any external funding. 

## Conflict of interest 

The authors declare no conflict of interest.
